# A Portable Micro-Gas Chromatography with Integrated Photonic Crystal Slab Sensors on Chip

**DOI:** 10.3390/bios11090326

**Published:** 2021-09-09

**Authors:** Priyanka Biswas, Chen Zhang, Yudong Chen, Zhonghe Liu, Seyedmohsen Vaziri, Weidong Zhou, Yuze Sun

**Affiliations:** Department of Electrical Engineering, University of Texas at Arlington, Arlington, TX 76019, USA; priyanka.biswas@mavs.uta.edu (P.B.); chen.zhang3@mavs.uta.edu (C.Z.); yudong.chen@mavs.uta.edu (Y.C.); zhonghe.liu@mavs.uta.edu (Z.L.); Seyedmohsen.vaziri@mavs.uta.edu (S.V.); wzhou@uta.edu (W.Z.)

**Keywords:** micro-gas chromatography, photonic crystals, volatile organic compounds, gas sensing, optofluidics

## Abstract

The miniaturization of gas chromatography (GC) systems has made it possible to utilize the analytical technique in various on-site applications to rapidly analyze complex gas samples. Various types of miniaturized sensors have been developed for micro-gas chromatography (µGC). However, the integration of an appropriate detector in µGC systems still faces a significant challenge. We present a solution to the problem through integration of µGC with photonic crystal slab (PCS) sensors using transfer printing technology. This integration offers an opportunity to utilize the advantages of optical sensors, such as high sensitivity and rapid response time, and at the same time, compensate for the lack of detection specificity from which label-free optical sensors suffer. We transfer printed a 2D defect free PCS on a borofloat glass, bonded it to a silicon microfluidic gas cell or directly to a microfabricated GC column, and then coated it with a gas responsive polymer. Realtime spectral shift in Fano resonance of the PCS sensor was used to quantitatively detect analytes over a mass range of three orders. The integrated µGC–PCS system was used to demonstrate separation and detection of a complex mixture of 10 chemicals. Fast separation and detection (4 min) and a low detection limit (ng) was demonstrated.

## 1. Introduction

Micro-gas chromatography (μGC) systems have shown immense growth in the last two decades [1,2,3,4,5]. It provides a less invasive option for gas analysis that can be monitored in real time and offer reliable quantitative data in essential sectors, including industry, healthcare, environment, and national security. The key attributes of a μGC system are its good compactness, automaticity, reduced power consumption, minimal maintenance costs, and suitability for field analysis. Thus, it provides the advantages of rapid analysis and turnaround time as well as simpler sample preparation. The miniaturization of a GC system calls for miniaturization of the detectors as well. The conventional detectors suffer from large dead volume and thus slow response time. Out of various transduction mechanisms used in μGC systems so far, optical sensing has emerged to be a popular mode of sensing modality due to its immunity to electromagnetic interference and cross talk, high sensitivity, ability to function in hazardous conditions, remote operation, and fast response. Some optical detectors used in µGCs are based on surface plasmon resonance [6], ring resonators [7,8,9,10], Fabry–Perot interferometry [11], diode laser spectroscopy [12], optomechanical sensors [13], and photoacoustic spectroscopy [14]. Localized surface plasmon resonance of a gold nanoparticle monolayer immobilized in a glass capillary has been demonstrated in the detection of volatile organic compounds (VOCs) with a detection limit ranging from 22 ng to 174 ng [6]. The peak width reported for the analytes were relatively broad (2.1 s–6.1 s) at a flow rate of 3.5 mL/min due to a relatively large volume of the sensor gas chamber compared to the size of the GC separation column. Although the sensor is compatible with the µGC system, with a high value of dead volume, it compromises the separation efficiency and is challenging for integration in a compact µGC system. Optofluidic ring resonator (OFRR) is another commonly used detector in µGC that combines vapor sensing and fluidic transport properties in one platform [7,8,9,10]. A polymer coated OFRR used in conjunction with µGC system detected multiple vapor species with a detection limit at the low ppm level. Later, the structure was modified and coated with nanoparticles to add detection selectivity by exploiting the wavelength dependence of VOC response. This system demonstrates the minimum detectable mass is 75 ng to 320 ng [9]. Recently, researchers have also attempted a combination of optical and mechanical transducer in a µGC system. The optomechanical sensor consists of a racetrack resonator, inside which light undergoes a phase shift due to the vibration of a mechanical resonator [13]. Thus, it exhibited a detection limit of ppm level of VOCs. Although all these works have reported reasonably moderate sensing performance in conjunction with the GC platform, it remains challenging to develop microsensors and nanosensors for a µGC system that are miniaturized, efficient in gas fluidics, easy to operate, and can be reliably fabricated on large scale with straightforward processes. 

Photonic crystals (PC) have adopted the inherent properties of optical sensors, along with compatibility with standard silicon IC fabrication for reliable mass production and chip integration. Its strong optical confinement and compact orientation makes it even more attractive in sensing applications [15,16,17,18,19,20,21]. The strong light–matter interaction in the PC sensor allows for the detection of dilute samples within a small interaction volume. The flat photonic bandgap and low group velocity of light in the periodic structure provides for a large interaction time. The lower group velocity of light in PC also gives higher enhancement factor [21]. These structures with defects (point or line) allow for even better light localization. Two dimensional defect-based PCs have been broadly explored for gas sensing in two main forms, PC cavity and PC waveguide [22,23,24,25]. PC cavity sensors are designed by creating either an acceptor or a donor type point defect or, alternatively, by establishing optical confinement in a small area through structural modification. They generally have a lower mode volume and are more effective in sensing a small quantity of sample in comparison to its counterpart. A Cryptophane E infiltrated defected PC side coupled cavity structure using fiber ring-down technology for demodulation of PC resonance spectrum with high precision was demonstrated by Qian et al. [26]. The simulation showed a refractive index sensitivity of 450 nm/RIU, quality factor (Q-factor) of 10^3^, and a methane detection limit of 2.37 ppm. The infiltrating molecule is only selective to methane and hence is not sensitive to any other gaseous analytes. Experimentation would have given a better estimate of its performance. Another silicon PC microcavity with air slot was fabricated and demonstrated showing a Q-factor of 32,900 and sensitivity of 421 nm/RIU [27]. The application for sensing nitrogen, hydrogen, and carbon dioxide experimentally revealed a detection limit of 1 × 10^−5^ RIU. If a line defect is found in the periodic structure of a PC, it is referred to as a PC waveguide. Here, the light is confined laterally by the PC and vertically by the total internal reflection. Hence, only a certain frequency range of light can be transmitted through the waveguide [28]. A slotted PC waveguide exhibited gas sensing capacity by selectively impregnating two rows of holes with different refractive index liquid [29]. This structure created on silicon-on-insulator (SOI) demonstrated a detection limit of 1.56 ppm (CO_2_) and was found to be sensitive to carbon monoxide, carbon dioxide, and hydrogen sulfide by adjusting the refractive index of the liquid to match the absorption peak of the analyte under investigation. Similar to the in-plane defect-based PC sensors, out-of-the-plane defect-free PC sensors have also been investigated. Due to periodic refractive index modulation in the PCS, guided modes exist for in-plane light manipulation, with wavelengths determined by the lattice parameters as well as the index modulation contrast. Similar to the guided mode, for guided resonance, the electromagnetic power is also strongly confined within the slab. Unlike the guided mode, however, the resonance can be coupled with external radiation (leaky mode above the light line). Therefore, guided resonance can provide an efficient way to channel light from within the slab to the external environment. A detailed theoretical analysis of guided resonances in 2D PCS was first presented by Fan et al. [30]. Due to its origin and the asymmetric resonance features, it falls into the general area of Fano resonances [31]. In comparison to the 2D defect-based PC, defect free PCS has the advantages of flexible alignment requirements, a straightforward fabrication process with more tolerance to deformation in the lattice structure, and adaptability to multiplexed operations. We have explored a defect free Si PCS on SOI for vapor sensing in our previous work [32]. A 57 ppm detection limit was established for hexane. However, the response time for the sensor was in the order of 100 s due to a large gas cell and added dead volume, which is inadequate for use as a real time detector in a µGC system. Although reasonable sensitivity and good Q-factor were demonstrated in PC gas sensors, PC inherently lacks detection specificity. Some of this research work has addressed the issue by modifying either the instrumentation or elements of the structure itself [26,29,33,34]. However, the process is labor intensive and prone to error. In some cases, the results are difficult to interpret. The selectivity issue can be solved using PC sensors together with a µGC. A mixture of gases flowing through a GC column separates, resulting in individual analytes arriving at the end of the column at a unique time (i.e., elution time). The real time response from a micro-detector placed at the end of the column can identify analytes specifically through the elution time and quantitatively through the signal intensity to reveal the analyte concentration. An ideal microsensor used in µGC should have negligible dead volume, efficient gas fluidics, and fast intrinsic response time, so that the detector induced peak broadening is minimal, and thus maintaining the separation resolution of the µGC system. Additionally, high sensitivity and large dynamic range are desirable features of a microsensor in various applications.

Here, we described the integration of a µGC with a 2D defect free PCS sensor through a transfer printing process and its application in separation and detection of complex VOC mixtures. To the best of our knowledge, this is the first work on the integration of photonic crystal sensors with µGC. An easy to implement cross polarization optical setup was used to couple the Fano resonance of a defect free PCS in free space. This avoids any coupling issue with optical fiber at the interface due to modal mismatch [35]. We first developed and assembled a portable µGC system that is fully automated using a LabVIEW program. Si PCS fabricated on SOI substrate was transfer printed using a PDMS stamp onto glass substrate and then anodically bonded to a silicon microfluidic channel without compromising efficient gas fluidics. The integrated system was used to separate and detect a mixture of 10 VOCs in under 4.5 min. We further transfer printed the PCS on a glass wafer and directly bonded to microfabricated Si GC columns. Separation and detection of 4 VOC mixture was demonstrated using an integrated Si µGC column and an on-column PCS sensor at room temperature.

## 2. Materials and Methods

### 2.1. Materials

Carbopack B (60–80 mesh) and Carbopack X (40–60 mesh) was purchased from Supelco (Bellefonte, PA). Analytical grade pentane, hexane, heptane, benzene, trichloroethylene, toluene, hexanal, chlorobenzene, ethylbenzene, p-xylene, and 2-heptanone were purchased from Sigma-Aldrich (St. Louis, MO, USA). The chemicals had purity greater than or equal to 99% and were used without any further modification. OV-101 was acquired from Ohio Valley Speciality Company (Marietta, OH, USA). Rtx-5MS (10 m × 0.25 mm ID, 0.25 µm coating thickness), universal press-tight glass capillary column connectors, angled Y-connectors, tedlar bags, and guard column were purchased from Restek Corporation (Belafonte, PA, USA). Quartz capillary (1.5 mm OD × 1.1 mm ID) were bought from Sutter Instrument Co. (Novato, CA, USA). The 2-port and 3-port solenoid valves were purchased from Lee Company (Westbrook, CT, USA). A diaphragm pump was purchased from Gast Manufacturing (Benton Harbor, MI, USA). A type-K thermocouple was purchased from Omega Engineering (Stamford, CT, USA). Kanthal and nickel wires were bought from Lightning Vapes (Bradenton, FL, USA). A data acquisition card (DAQ card), USB-6003 (16 bits) was purchased from National Instruments (Austin, TX, USA). Silicon wafers were acquired from University Wafer Inc (South Boston, MA, USA). A customized printed circuit board (PCB) was designed and manufactured by M.A.K.S., Inc. (Troy, MI, USA). A 36 V AC/DC converter was bought from TDK-Lambda Americas Inc. (National City, CA, USA). A 12 V and 24 V DC/DC converter was purchased from CUI Inc (Tualatin, OR, USA). A miniature helium cartridge was purchased from Leland Gas Tech (South Plainfield, NJ, USA). All optical components were procured from Thorlabs (Newton, NJ, USA) and Edmund Optics (Barrington, NJ, USA).

### 2.2. Sample Preparation

To evaluate the integration of the µGC system with the PCS sensor, 11 polar and non-polar VOCs were used. Heptane, along with three other VOCs from this list, was used to show isothermal separation and detection using a Si µGC column and an on-column PCS detector. The chemical and physical properties of the chemicals are summarized in Table 1 [36,37,38,39,40]. The mixture was formulated by mixing equal volumes of all the analytes in their liquid form. A certain volume of the liquid mixture was then injected into a 3 L tedlar bag filled with helium using a gas tight syringe. The injected liquid was vaporized in the bag to form the test vapor. To prepare a sample of concentration below 10 µg/L, the analytes were first dissolved in pentane and then injected into the tedlar bag. For the high concentration sample (above 40 µg/L), the required volume of the analyte was injected into the tedlar bag directly.

### 2.3. Fabrication and Assembly of µGC

**Figure 1:**
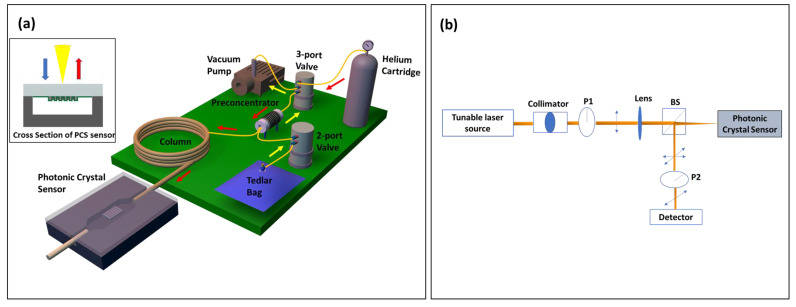
(**a**) Schematic of integrated µGC–PCS gas analysis system. The portable µGC system is packed into a small briefcase of 22” × 14” × 6.5” in size. The operation of µGC is controlled by a homemade LabVIEW code. The yellow (red) arrow shows the flow direction during the sampling (analyzing) stage. The PCS sensor is transfer printed to a glass substrate and then anodically bonded to a silicon microfluidic channel. The inset shows the cross-section view of the PCS sensor in a microfluidic channel. (**b**) Schematic of the optical characterization setup. BS: Beam Splitter, P1/P2: Linear Polarizer.

The µGC system was assembled using miniatured components, most of which were sourced off the shelf, except for preconcentrator, which was fabricated in house. All the components were electrically connected to the custom designed PCB board and controlled using a homemade LabVIEW program. Figure 1a shows the schematic of the µGC system, which is a completely free-standing system that can be carried around in a 22” × 14” × 6.5” hard plastic box. Sampling was carried out by the mini vacuum pump that pulled the analytes from the tedlar bag through the preconcentrator at a flow rate of 5 mL/min, allowing them to accumulate there. After the sampling period, helium flowed through the preconcentrator to remove any VOCs that failed to get adsorbed. Then, the analyzing phase started. At the beginning of this phase, the preconcentrator was heated using a combination of 36 V and 12 V power sources to reach 330 °C in less than 1.7 s to release a sharp peak of analyte mixture that was injected into the column by the carrier gas helium. The preconcentrator was constructed using a 3.3 cm long quartz capillary with a 1.5 mm OD and 1.1 mm ID. Carbopack B and Carbopack X were used in sequence in the sampling path to trap the analytes with vapor pressure ranging from 0.01 Torr to 95 Torr [41]. The preconcentrator was heated by resistive heating by tightly wrapping Kanthal wire around the capillary. In all measurements, a helium flow rate of 1 mL/min was used. Additionally, 10 m of Rtx-5MS column was employed for all temperature programmed separation, while a 3 m OV-101 coated on the microfabricated silicon µGC column was used for room temperature separation. For temperature programmed separation, the column temperature was initially set at 30 °C and then ramped at 30 °C/min to 90 °C. It was held at the final temperature for another 2 min. After separation, the analytes were detected using a 2D PCS free space coupled sensor, which was connected to the GC column by a 10 cm guard column. The optical setup used for detection has been described in detail in our previous publications [32,42]. Briefly, as illustrated in Figure 1b, a tunable laser source was focused on the PCS sample through a collimator, linear polarizer (P1), and lens under surface normal incidence condition. A beam splitter at the end of this optical path split the reflected light and allowed it to pass through another polarizer (P2), which has a transmission axis orthogonal to that of the polarizer P1, and finally to a photodiode. This cross-polarization arrangement blocked the background incident light and revealed the symmetry-protected bound states in the continuum (BIC) modes that are used for sensing [42,43].

## 3. Results

### 3.1. PCS Sensor Design, Fabrication, and Characterization

**Figure 2:**
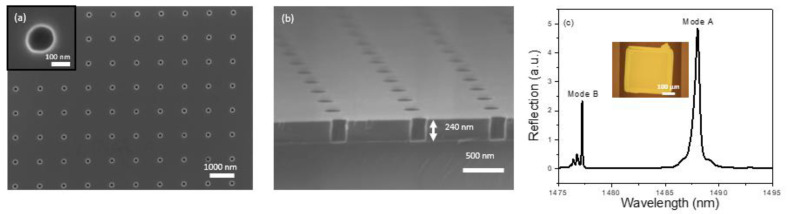
Characterization of a PCS sensor on SOI substrate and glass substrate. (**a**) Top view SEM image of the fabricated PCS sensor on SOI showing device parameters of a = 976 nm, r = 75 nm, t = 240 nm. The inset shows the top view for a single air hole in the PCS. (**b**) Cross sectional view SEM image of the PCS sensor on SOI. (**c**) Measured reflection spectrum of the PCS sensor on a glass substrate after transfer printing. Inset shows a microscope image of the PCS after transferring in a microfluidic channel.

The design of the 2D PCS was done using Stanford Stratified Structure Solver (S^4^) software package [44]. According to our previous studies [32,42,43], Si PCS with lattice constant, a = 976 nm, air hole radius, r = 75 nm, and Si slab thickness, t = 240 nm leads to a center wavelength of Fano resonance around 1500 nm and presents a detection limit around 10^−5^–10^−6^ RIU. The PCS was first fabricated on a SOI substrate using e-beam lithography (EBL) and reactive-ion etching (RIE) [45]. Scanning electron micrograph (SEM) images of the fabricated device are presented in Figure 2a,b.

Transfer printing is then used to transfer the PCS from the SOI substrate to a borofloat glass substrate [31,46,47,48]. In short, after patterning the Si PCS membrane on the SOI substrate, the sample is immersed into the hydrofluoric acid solution to etch the buried oxide. Once the sacrificial oxide layer is removed, the suspended PCS is picked up with a PDMS stamp. The peeling back is done quickly to overcome the bonding forces between the PCS membrane and the donor substrate. On successful removal of the PCS membrane from the Si surface, it is applied onto a borofloat glass substrate. The PCS membrane bonds with the receiver substrate with van der Waal’s force. To have a well printed sample, the peeling speed during pick up should be fast, while the printing speed during the release of the membrane should be slow. That is why this kind of printing is also known as kinetically controlled transfer printing [46]. It is worth noting that, over the years, the transfer printing process has improved dramatically, from the initial hand-based process to current automatic printing process with high yield (>99%) and high precision (~1 mm alignment accuracy) [49]. High performance optical devices achieved by the transfer printing technique have been demonstrated, including lasers [48] and heterogeneously integrated III-V/Si platforms for silicon photonics [50]. The glass with the transferred PCS is then anodically bonded to a microfluidic channel (width 500 µm, length 900 µm, and depth 120 µm) fabricated on a silicon chip using deep reactive ion etching (DRIE). Figure 2c shows the reflection spectrum of the PCS after transferring onto a glass substrate and a microscope image of the transferred PCS in the silicon microfluidic channel. The symmetry protected BIC mode (Mode A and Mode B in Figure 2c) can be revealed by the cross-polarization measurement, which is caused by a small inherent angle in the incident beam [42]. Both Mode A and Mode B can be used to track the spectral shift caused by chemical vapors and both modes present the same sensitivity [43]. Here, Mode B is selected for subsequent sensing experiments because it has a higher Q-factor than that of Mode A, and thus a better detection limit can be achieved in vapor sensing.

**Figure 3:**
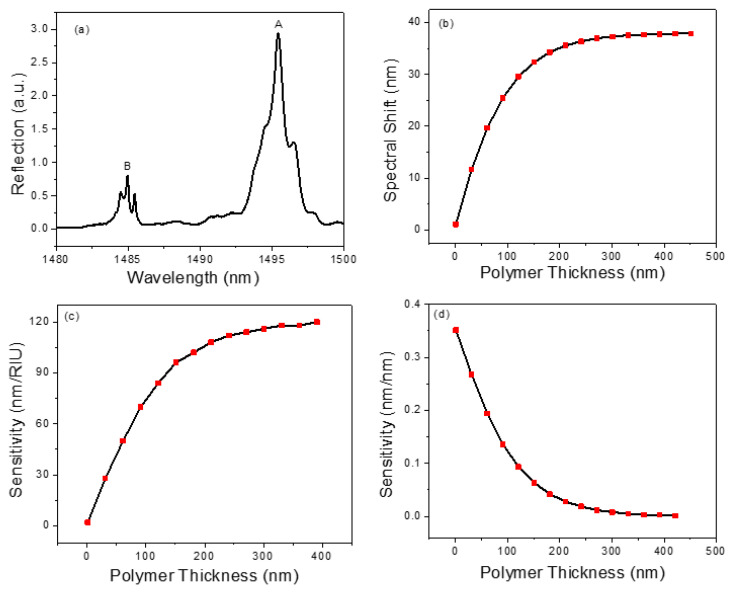
(**a**) Measured reflection spectrum of the transferred PCS sensor after coating. (**b**) Simulated spectral shift of PCS sensor for different polymer thicknesses. (**c**) RI sensitivity for different polymer thicknesses. (**d**) Thickness sensitivity for different polymer thicknesses.

In order to detect chemical vapors, a thin layer of polymer OV-101 (refractive index n = 1.4) is coated on the PCS surface through the static coating process [51,52]. The microfluidic channel housing the PCS is filled with a coating solution concentration of 2.5 mg/mL and the solvent is then removed using a vacuum overnight, leaving a thin layer of polymer on the PCS surface. After coating, the spectral position of the resonance mode in the reflection spectrum presents a red shift due to an increase in the effective refractive index experienced by the resonance mode. The reflection spectrum of the PCS after the coating process is presented in Figure 3a. To estimate the coating thickness on the PCS sensor, the relationship between spectral shift and polymer thickness is simulated and shown in Figure 3b. As the polymer thickness increases, the spectral shift increases linearly for the thickness below 60 nm and then gradually saturates. The slope of the resonance shift in relation to polymer thickness in the linear region is 0.33 nm/nm. Compared to the spectrum shown in Figure 2c, a red shift of 7.5 nm is observed by Mode B in the reflection spectrum. This corresponds to a 20 nm polymer thickness according to the simulation result shown in Figure 3b. After coating, the Q-factor of Mode B degrades from 16,000 to 9000, which is expected due to nonuniformity and surface texture of the coating.

On exposure to vapor molecules, the polymer layer on the PCS sensor swells up and experiences a change in the refractive index. This causes a spectral shift in the resonance mode of the PCS according to the relation below [53]:

(1) λ=(∂λ/∂n)·∆n+(∂λ/∂t)·∆t

where n and t are refractive index and thickness of the polymer, respectively. $S_{RI}=\partial \lambda /\partial n$ and $S_{t}=\partial \lambda /\partial t$ represents the RI sensitivity and thickness sensitivity of the optical mode under investigation, respectively. The RI sensitivity and thickness sensitivity for different polymer thickness are calculated and presented in Figure 3c,d, respectively. The RI sensitivity increases with the rise in polymer thickness, whereas the thickness sensitivity presents an opposite trend. For PCS coated with a thicker polymer layer (e.g., 200 nm), the detection sensitivity is primarily dominated by the RI sensitivity. However, for a thinner polymer coated PCS (e.g., 20 nm), both RI sensitivity and thickness sensitivity contribute to the sensing signal. To quantitatively measure the vapor concentration eluted in a µGC system, the sensor’s time response is a vital parameter to maintain the separation resolution achieved by the µGC. For this reason, a thin layer of polymer coating on the PCS sensor is preferred, because the time required for the vapor molecules to diffuse in and out of the polymer is sufficiently small. Therefore, in this study, a polymer layer of 20 nm is used in the experiments.

### 3.2. Sensitivity and Detection Limit Characterization

**Figure 4:**
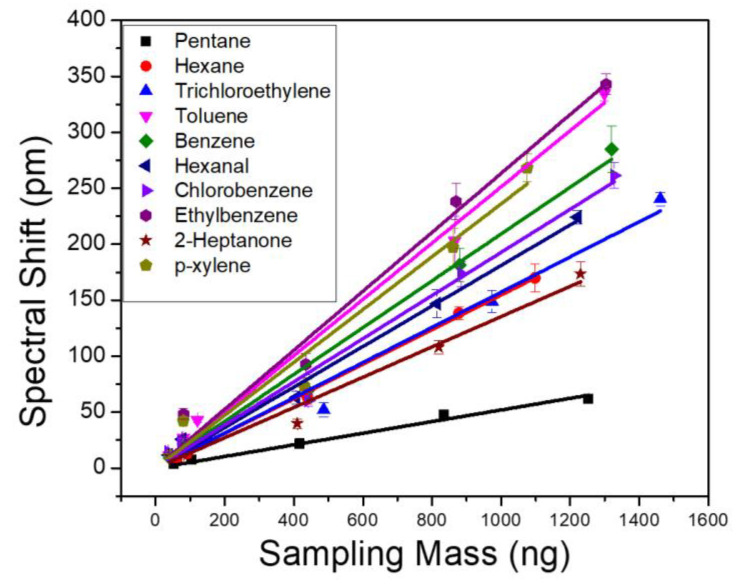
PCS sensor spectral shift obtained as a function of sampling mass for 10 VOCs. All analytes are measured with the same column temperature ramping profile and a flow rate of 1 mL/min.

To evaluate the sensor performance, we first characterize the sensitivity of VOCs by injecting individual analytes to the µGC system and monitoring the PCS resonance spectral shift in realtime. A tedlar bag containing a predefined concentration of analyte was connected to the µGC setup. Based on the sampling time and flow rate of the vacuum pump, a fixed mass of the analyte accumulates in the dual adsorbent preconcentrator. The sampling flow rate was 5 mL/min and sampling time was varied to achieve a sampling mass over three orders of magnitude, between 0–1500 ng. The calibration curves for each of the 10 VOCs are shown in Figure 4. Each solid line in Figure 4 represents linear fitting of the five-point raw data, which was used to calculate the sensitivity of the analytes. The slope of the fitted line represents the analyte sensitivity in pm/ng. The sensitivity of the analytes is also outlined in Table 2. The *R*^2^ values for all the fitted lines were greater than 0.98. Linearity of the PCS spectral shift is also examined for the 10 VOCs. The response of the PCS sensor to all the analytes were highly linear, over three orders of magnitude of the sampling mass. Ethylbenzene and toluene exhibited the highest sensitivity, while pentane showed the lowest. Since OV-101 is a non-polar polymer, it is supposed to interact with non-polar analytes, such as ethylbenzene and toluene, more and thus have higher sensitivity. However, polarity is not the only factor that should be considered here. The fraction of the analyte that stays within the polymer in equilibrium with the carrier gas on top of it is also a determining factor. This fraction is determined by the partition coefficient of the polymer for a specific analyte as shown in Table 1. The higher the partition coefficient, the more the analyte tends to stay in the polymer layer rather than in the mobile phase. Pentane, in this case, has the lowest partition coefficient among the 10 analytes, which causes it to have a lower sensitivity compared to the other analytes. The limit of detection (LoD) for each of the VOC is calculated using:

(2) $$LoD=\frac{3\sigma }{S}$$

where 3σ is the three standard deviations of the system noise, and S is the measured analyte sensitivity shown in Table 2. The detection limit of the PCS sensor for the 10 analytes ranges from 7 ng to 36 ng.

### 3.3. Analyte Separation

**Figure 5:**
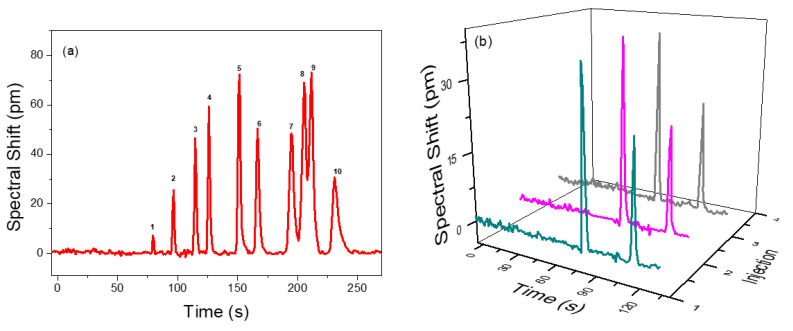
(**a**) Temperature programmed chromatogram for a VOC panel consisting of 10 VOC in a 10 m Rtx-5MS column with helium as the carrier gas flowing at 1 mL/min. Details of the identified peaks are provided in Table 3. (**b**) Repeatability of the separation demonstrated with a mixture of benzene (82 ng) and pentane (776 ng) under the same conditions over three consecutive injections.

To demonstrate the separation performance of the integrated µGC and PCS sensor system, a mixture of 10 VOCs was prepared in a tedlar bag and was tested using a 10 m long Rtx-5MS column under the temperature programed condition. The separation and detection of 10 VOCs are shown in Figure 5a. All the peaks are symmetric with peak width (FWHM) less than 5.1 s. All analytes are well separated within 4 min, except ethylbenzene and p-xylene, which have partial overlap due to having very close boiling points and similar polarities, which is quite difficult to fully resolve using the Rtx-5MS column [54,55,56]. They are both non-polar and have similar partition factors. A reduced temperature would have resolved them, but the analyzing time would be increased. Therefore, a trade-off between separation and overall analysis time is required. The elution time could be further reduced using a higher flow rate or a shorter column at the expense of separation capacity. Details of the analyte mixture and their peak width (FWHM) and spectral shift are presented in Table 3. The sensing signal fully returns to baseline once the VOCs pass through, which demonstrates that the polymer in PCS is rapidly and completely regenerated, as are the efficient gas fluidics of the integrated µGC and PCS sensor system.

Figure 5b describes the repeatability and reliability of analyte sampling/injection, separation, and detection achieved by the µGC–PCS sensor system. A mixture of benzene and pentane is pumped into the µGC system over three consecutive injections. The retention time, peak width, and spectral shift were reproduced with high precision. The standard deviation on the spectral shift of pentane and benzene is less than 4%.

### 3.4. Analyte Separation and Detection with a PCS in Microfabricated GC Column

**Figure 6:**
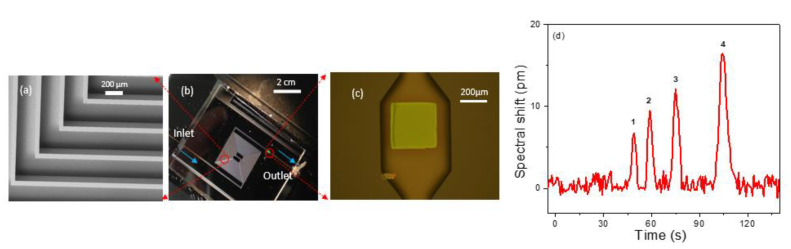
(**a**) Angled view SEM image of a Si column. (**b**) An image of the 3 m Si column bonded with glass with PCS (green dot) at the outlet of the column and blue arrow showing the flow direction. (**c**) Microscope image of the transferred PCS aligned to the expansion chamber at the outlet of the column. (**d**) Chromatogram for separation of hexane, heptane, benzene, and toluene in a 3 m on chip silicon column with helium carrier gas at 1 mL/min at room temperature. Sampling mass and FWHM for the analytes are: 1. Hexane (220 ng) (3.15 s), 2. Benzene (293 ng) (3.50 s), 3. Heptane (226 ng) (4.24 s), 4. Toluene (289 ng) (5.53 s).

We also performed on-chip separation and detection by transferring the PCS sensor at the end of a microfabricated silicon column. A 3 m long rectangular cross section spiral channel with a 250 µm width and 120 µm depth was created on silicon substrate using the DRIE process [57]. The PCS was transfer printed to a predefined location on a borofloat glass substrate and subsequently aligned to the expansion chamber located at the column outlet, with the PCS facing the column. Then, the silicon column is anodically bonded to the borofloat glass substrate to seal off the open surface of the column. Fused silica capillary glued to the ends of the silicon channel serves as inlet and outlet of the chip. An angled SEM image of the microfabricated column is presented in Figure 6a, which shows the section of column in the red circle in Figure 6b. The integrated micro-column/PCS with inlet and outlet capillaries is shown in Figure 6b. Figure 6c illustrates the PCS sensor sitting at the outlet of the column in an expansion chamber of width 500 µm, length 900 µm, and depth 120 µm. Subsequently, the column was coated with OV-101 polymer along with the PCS sensor using static coating method resulting in a stationary phase nominal thickness of 100 nm. The column was characterized using a mixture of four analytes: hexane, heptane, benzene, and toluene. The flow rate for the carrier gas, helium, was set at 1 mL/min, and the process was performed under isothermal conditions at room temperature. Analyte mass ranging from 200–300 ng was sampled within 4 min, and the analysis was completed in another 2 min. The peak width for the analytes varies from 3 to 5.5 s. Figure 6d shows the chromatogram for the on-column detection.

## 4. Conclusions

We have developed a compact, fast, and user-friendly µGC system with integrated free space coupled 2D PCS sensor for analyzing a wide spectrum of VOCs. Transfer printing of the PCS on glass substrate and bonding to a microfluidic gas cell or microfabricated GC column ensures efficient gas fluidic integration with the µGC system. A µGC system enabled with PCS sensor fulfills the requirement of a portable, nondestructive, universal vapor detection system with flexibility in design and long lifetime. The defect free PCS sensor uses the spectral shift in its intrinsic Fano resonance, caused by adsorption of vapor molecules onto the sensing polymer, to quantitatively measure VOCs of different variety.

The simulation results of RI and thickness sensitivity of the PCS have been reported. The PCS sensor’s RI and thickness sensitivity show an opposite trend, with an increase in the polymer thickness. The sensitivity can be further optimized by engineering PCS structures to have an increased resonance mode of optical field distribution in the thin layer of polymer. The spectral shift in the PCS sensor exhibited a linear relationship with the concentration of the analyte over a mass range of three orders. The detection limit obtained from the calibration curves of individual analyte were in the order of low ng. The integrated system showed complete separation and detection for a complex mixture of 10 chemicals in 4 min. Fast separation and detection were demonstrated by a PCS sensor integrated with the on chip microfabricated column. Two dimensional PCS sensors can easily be mass produced by MEMS fabrication with high reproducibility. Future work involves further miniaturization of the µGC system by fabricating all components in silicon, integrating the light source and detector with PCS sensors, and integrating the system to develop a complete gas analyzer on a chip.

## Figures and Tables

**Table 1:** Characteristics of the analytes at room temperature.

Chemical Name	Vapor Pressure (Torr)	Partition Coefficient	Polarity
Pentane	434	82	Nonpolar
Hexane	132	215	Nonpolar
Heptane	53.3	565	Nonpolar
Benzene	95	296	Nonpolar
Trichloroethylene	47	981	Polar
Toluene	28.5	815	Nonpolar
Hexanal	10	2300	Polar
Chlorobenzene	11.8	2494	Polar
Ethylbenzene	10	2020	Nonpolar
p-xylene	9	2220	Nonpolar
2-Heptanone	3.9	3617	Polar

**Table 2:** Sensitivity and detection limit characterization for a panel of 10 VOCs.

Chemical Name	Sensitivity (pm/ng)	Standard Error	*R* ^2^	LOD (ng)
Pentane	0.05198	0.00189	0.99343	36.36
Hexane	0.15463	0.00182	0.99931	12.22
Benzene	0.20916	0.00768	0.9933	9.04
Trichloroethylene	0.15724	0.00757	0.98852	12.02
Toluene	0.25129	0.00702	0.99611	7.52
Hexanal	0.18107	0.0056	0.99524	10.44
Chlorobenzene	0.19274	0.00838	0.99062	9.81
Ethylbenzene	0.2633	0.01101	0.99132	7.18
p-xylene	0.23611	0.01369	0.98341	8.00
2-Heptanone	0.13536	0.0059	0.99057	13.96

**Table 3:** Separation performance of the VOC mixtures in Figure 5.

Peak #	Chemical	Sampling Mass (ng)	FWHM (s)	Spectral Shift (pm)
1	Pentane	209	1.3	7.2
2	Hexane	220	1.8	25.6
3	Benzene	293	1.9	46.5
4	Trichloroethylene	488	1.8	59.3
5	Toluene	289	2.4	72.2
6	Hexanal	271	2.8	50.4
7	Chlorobenzene	369	4.1	48.5
8	Ethylbenzene	290	3.6	68.9
9	p-xylene	287	4.0	73.0
10	2-Heptanone	273	5.1	30.7

## Data Availability

The data presented in this study are available upon reasonable request from the corresponding author.
